# Supplementary material for: Hybrid Models and Biological Model Reduction with PyDSTool
Source: PLoS Comput Biol. 2012 Aug 9;8(8):e1002628. doi: 10.1371/journal.pcbi.1002628 (PMC3415397; doi:10.1371/journal.pcbi.1002628)
Supplement: Text S4 — Complete source code for the PyDSTool package (version 0.88.120504). Includes API documentation and help files linking to web pages. This file is identical to the current public release on Sourceforge.net. (ZIP) [file pcbi.1002628.s004.zip › PyDSTool/html/PyDSTool.scipy_ode.ode-class.html]

xml version="1.0" encoding="ascii"?


PyDSTool.scipy\_ode.ode


| Home | Trees | Indices | Help | PyDSTool |
| --- | --- | --- | --- | --- |

Package PyDSTool ::
Module scipy\_ode ::
Class ode

[hide private]

[frames] | no frames]

# Class ode

source code

```
object --+
         |
        ode
```

---

```
ode  - a generic interface class to numeric integrators. It has the
following methods:
  integrator = ode(f,jac=None)
  integrator = integrator.set_integrator(name,**params)
  integrator = integrator.set_initial_value(y0,t0=0.0)
  integrator = integrator.set_f_params(*args)
  integrator = integrator.set_jac_params(*args)
  y1 = integrator.integrate(t1,step=0,relax=0)
  flag = integrator.successful()

Typical usage:
  r = ode(f,jac).set_integrator('vode').set_initial_value(y0,t0)
  t1 = <final t>
  dt = <step>
  while r.successful() and r.t < t1:
      r.integrate(r.t+dt)
      print r.t, r.y
where f and jac have the following signatures:
  def f(t,y[,arg1,..]):
      return <f(t,y)>
  def jac(t,y[,arg1,..]):
      return <df/dy(t,y)>
```


|  |  |
| --- | --- |
| Instance Methods | [hide private] |

|  |  |
| --- | --- |
| \_\_init\_\_(self, f, jac=None)  Define equation y' = f(y,t) where (optional) jac = df/dy. | source code |

|  |  |
| --- | --- |
| set\_initial\_value(self, y, t=0.0)  Set initial conditions y(t) = y. | source code |

|  |  |
| --- | --- |
| set\_integrator(self, name, \*\*integrator\_params)  Set integrator by name. | source code |

|  |  |
| --- | --- |
| integrate(self, t, step=0, relax=0)  Find y=y(t), set y as an initial condition, and return y. | source code |

|  |  |
| --- | --- |
| successful(self)  Check if integration was successful. | source code |

|  |  |
| --- | --- |
| set\_f\_params(self, \*args)  Set extra-pars for user-supplied function f. | source code |

|  |  |
| --- | --- |
| set\_jac\_params(self, \*args)  Set extra-pars for user-supplied function jac. | source code |

**Inherited from `object`**:
`__delattr__`,
`__getattribute__`,
`__hash__`,
`__new__`,
`__reduce__`,
`__reduce_ex__`,
`__repr__`,
`__setattr__`,
`__str__`


|  |  |
| --- | --- |
| Properties | [hide private] |

**Inherited from `object`**:
`__class__`


|  |  |
| --- | --- |
| Method Details | [hide private] |

|  |  |
| --- | --- |
| \_\_init\_\_(self, f, jac=None)  *(Constructor)* | source code |

```
Define equation y' = f(y,t) where (optional) jac = df/dy.
User-supplied functions must have the following signatures:
def f(t,y,...):
    return <f(t,y)>
def jac(t,y,...):
    return <jac(t,y)>
where ... means extra pars that can be set with
  set_(f|jac)_params(*args)
methods.
```

Overrides: object.\_\_init\_\_

  


| Home | Trees | Indices | Help | PyDSTool |
| --- | --- | --- | --- | --- |

|  |  |
| --- | --- |
| Generated by Epydoc 3.0.1 on Fri May 4 15:24:10 2012 | http://epydoc.sourceforge.net |
